# Factors associated with smoking among tuberculosis patients in Spain

**DOI:** 10.1186/s12879-016-1819-1

**Published:** 2016-09-14

**Authors:** María Ángeles Jiménez-Fuentes, Teresa Rodrigo, María Neus Altet, Carlos A. Jiménez-Ruiz, Martí Casals, Antón Penas, Isabel Mir, Segismundo Solano Reina, Juan Antonio Riesco-Miranda, Joan A. Caylá, Smoking and Tuberculosis Research Working Group, R. Agüero, J. L. Alcázar, N. Altet, L. Altube, F. Álvarez Navascués, L. Anibarro, M. Barrón, P. Bermúdez, R. Blanquer, L. Borderías, A. Bustamante, J. L. Calpe, J. A. Caminero, F. Cañas, F. Casas, X. Casas, E. Cases, R. Castrodeza, J. J. Cebrián, J. E. Ciruelos, A. E. Delgado, M. L. De Souza, D. Díaz, M. Dominguez Alvarez, B. Fernández, A. Fernández, J. Gallardo, M. Gallego, M. M. García Clemente, C. García, F. J. García, F. J. Garros, A. Guerediaga, J. A. Gullón, C. Hidalgo, M. Iglesias, G. Jiménez, M. A. Jiménez-Fuentes, J. M. Kindelan, J. Laparra, R. Lera, T. Lloret, M. Marín, J. T. Martínez, E. Martínez, A. Martínez, J. F. Medina, C. Melero, C. Milà, I. Mir, C. Morales, M. A. Morales, V. Moreno, A. Muñoz, L. Muñoz, C. Muñoz, J. A. Muñoz, I. Parra, A. Penas, J. A. Pérez, P. Rivas, J. Rodríguez, J. Ruiz-Manzano, J. Sala, M. Sánchez, P. Sánchez, F. Sanz, M. Somoza, E. Trujillo, E. Valencia, A. Vargas, I. Vidal, R. Vidal, M. A. Villanueva, A. Villar, M. Vizcaya, M. Zabaleta, G. Zubillaga

**Affiliations:** 1 Unidad de Tuberculosis Valle de Hebrón-Drassanes. Programa Especial de Enfermedades Infecciosas, Hospital Universitario Valle de Hebrón, Av Drassanes, 17-21, 08001 Barcelona, Spain; 2 Programa Integrado de Investigación en Tuberculosis (PII TB) de la Sociedad Española de Neumología y Cirugía Torácica (SEPAR), Barcelona, Spain; 3 Unidad de Investigación de Tuberculosis de Barcelona, Barcelona, Spain; 4 Fundación Respira de la SEPAR, Barcelona, Spain; 5 Centro de Investigación Biomédica en Red de Epidemiología y Salud Pública (CIBERESP), Madrid, Spain; 6 Serveis Clínics S.A., Barcelona, Spain; 7 Unidad Especializada de Tabaquismo de la Comunidad de Madrid, Madrid, Spain; 8 Servicio de Epidemiología de la Agencia de Salud Pública de Barcelona, Barcelona, Spain; 9 Hospital Lucus Augusti, Lugo, Spain; 10 Hospital Son Llàtzer, Palma de Mallorca, Spain; 11 Hospital Gregorio Marañón, Madrid, Spain; 12 Complejo Hospitalario de Cáceres, Cáceres, Spain; 13 Centro de Investigación Biomédica en Red de Enfermedades Respiratorias (CIBERES), Madrid, Spain; 14 Departamento de Salud Pública, Universitat Autónoma de Barcelona, Barcelona, Spain

**Keywords:** Tuberculosis, Smoking, Predictors, Prevention

## Abstract

**Background:**

To determine the prevalence of smoking and analyze associated factors in a cohort of patients diagnosed with tuberculosis (TB) in Spain between 2006 and 2013.

**Methods:**

Multicenter, cross-sectional, descriptive, observational study using a national database of TB patients, using logistic regression to calculate odds ratios (OR) and confidence intervals (CI).

**Results:**

We analyzed 5,846 cases (62 % men, mean age 39 years, 33 % foreigners). 23.4 % were alcohol abuser, 1.3 % were injected drug users (IDU), 4.6 % were co-infected with HIV, and 7.5 % had a history of TB treatment. 6.6 % and 0.8 % showed resistance to one and multiple drugs, respectively. The predominant clinical presentation was pulmonary (71 %) with a cavitary radiological pattern in 32.8 % of cases. 82 % of cases were confirmed microbiologically, and 54 % were smear-positive microscopy.

2,300 (39.3 %) patients were smokers. The following factors were associated with smoking: male sex (OR = 2.26;CI:1.97;2.60), Spanish origin (OR = 2.79;CI:2.40–3.24), alcoholism (OR = 2.85;CI:2.46;3.31), IDU (OR = 2.78;CI:1.48;5.52), homelessness (OR = 1.99;CI:1.14–3.57), pulmonary TB (OR = 1.61;CI:1.16;2.24), cavitary radiological pattern (OR = 1.99;CI:1.43;2.79) and a smear-positive microscopy at the time of diagnosis (OR = 1.39;CI:1.14;1.17).

**Conclusions:**

The prevalence of smoking among TB patients is high. Smokers with TB have a distinct sociodemographic, clinical, radiological and microbiological profile to non-smokers.

## Background

Smoking and tuberculosis (TB) are two of the biggest public health problems worldwide [1]. Smoking is one of the leading preventable causes of premature death, producing 6 million deaths a year. At present, about 33 % of the world population smokes, mainly in countries with a high prevalence of TB. In turn, TB causes 9.6 million incident cases and 1.5 million deaths in 2014 [2, 3].

While, the relationship between smoking and TB has been recognized for almost a century, the impact of smoking on TB has only been demonstrated in last decade [4–7]: both active and passive exposure to smoke are independent risk factors for TB infection [8, 9], the progression of TB infection to disease [10, 11], greater disease severity, and increased risk of post-treatment relapse and mortality [12–14].

A recent study based on mathematical modelling estimated that, between 2010 and 2050, smoking could raise the number of TB cases worldwide by 18 million, and substantially increase secondary mortality if current trends in tobacco consumption are maintained [15]. The World Health Organization and the International Union Against Tuberculosis and Lung Disease issued regulations in 2007 to control these two clearly related epidemics [1].

Smoking prevalence among TB patients could be higher than the objectified general population in many countries; in China a case-control study shows prevalence of 54.6 % [16], in South Africa found that 56 % of people with active TB were smokers [17], a study in rural India found that 81.5 % of TB cases had previously smoked at some time in their life [18] and in Georgia the prevalence of current smokers among the diagnoses of TB represents 45.9 % [19]. Currently, there are no reliable data on the prevalence of smoking among TB patients in Spain, with the exception of one study carried out in Catalonia between 1996 and 2002, which reported a smoking prevalence of 34.9 % [12].

The aim of our study was to determine the prevalence of smoking among patients diagnosed with TB in Spain between 2006 and 2013, and to identify factors associated with smoking in this population. The ultimate goal is to design assistance and support strategies for smoking cessation to improve clinical outcomes, reduce TB transmissibility, and improve prognosis and survival.

## Methods

We performed a multicenter, cross-sectional, descriptive, analytical and observational study.

We included patients diagnosed with TB between 1 January 2006 and 31 December 2013 within the catchment area of the Integrated TB Research Program (PII TB) Working Group of the Spanish Society of Pneumology and Thoracic Surgery (SEPAR) in 60 centers in Spanish Autonomous Communities.

We included patients aged >18 years with a diagnosis of TB, as determined by: 1) positive smear, or negative smear with positive culture for *Mycobacterium tuberculosis*, or extra-pulmonary TB as demonstrated by granulomas tubercular on histology; 2) patients suspected TB (clinical, radiological, epidemiological and/or laboratory results) with a good response to TB treatment without other diagnosis.

Patients were considered to be smokers if they reported having smoked ≥1 cigarettes per day continuously during the year preceding the diagnosis of TB and no smoker the person who has smoked less than 100 cigarettes in his life [20].

The information is collected by clinical researcher from the interview and review of the history in three visits: at the time of diagnosis, the second month and the end of treatment. For all cases, we collected socio-demographic data, living status, origin (native or immigrant), place of diagnosis, alcohol consumption (men consuming over 280 g alcohol per week, an women over 168 gr, were considered alcoholics), use of intravenous heroin or/and cocaine drugs (IDU), delayed diagnosis (>50 days), TB localization, radiology, microbiology results and sensitivity study, history of TB, HIV infection, clinical progression, drug treatment, and treatment outcome (correct: cured, treatment completed; incorrect: therapeutic failure, moved away/transferred, lost to follow-up and death).

The information obtained from each patient was stored in an electronic data collection notebook (DCN) implemented in a software application available to each study investigator via a personal identifier and password.

The study was conducted according to the requirements of the Declaration of Helsinki and Spanish Data Protection Law 15/1999. All patients gave their informed consent to participate in this study, which was approved by the Clinical Research Ethics Committee of Vall d'Hebron University Hospital Foundation - Research Institute.

### Statistical analysis

We performed a descriptive study of the prevalence of smoking among participants, and the frequency distribution of other variables. We performed bivariate analysis of factors potentially associated with smoking (yes/no) by comparing proportions between groups using the *χ*^2^ test. Results with p < 0.05 were considered statistically significant. For the multivariable analysis, we fit logistic regression models using the backward stepwise selection method to include variables that were relevant to the study, as well as those with p < 0.001 in the bivariate analysis, and to compute Odds Ratios (OR) and 95 % Confidence Intervals (CI). We used the Hosmer-Lemeshow test to evaluate the goodness of fit of each model. All analysis was performed using IBM SPSS Statistic19.

## Results

We included 5,846 TB patients; with a mean age of 39 years (range 18–100 years), 3,626 (62 %) men, and 1,941 foreigners (33 %). 23.4 % were pathological alcohol drinkers, and 1.3 % were IDU. 4.6 % of cases were coinfected with HIV, 438 (7.5 %) had a history of TB treatment, and 49.5 % were diagnosed by hospital emergency services. 6.6 % of cases were found to be resistant to any drug, and 0.8 % to multiple drugs. The predominant clinical presentation was pulmonary tuberculosis (4,149 cases, 71 %), with a cavitary radiological pattern in 32.8 % of cases. 82 % of cases were confirmed microbiologically, of which 54 % were smear-positive.

**Fig. 1:**
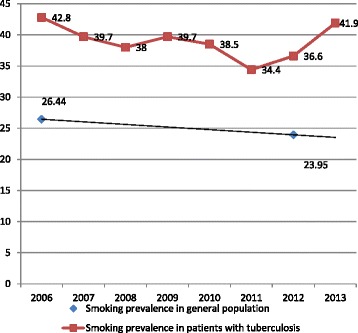
Trend in the prevalence of smoking in the general population and among smokers with tuberculosis 2006–2013

A total of 2,300 (39.3 %) patients were smokers, and this proportion remained stable during the course of the study Fig. 1.

The bivariate analysis to detect factors associated with smoking (Table 1) indicated a higher prevalence of smoking among men, Spanish nationals, and individuals who live alone or in confinement, or who are homeless, regardless of age or employment status. Smoking was also associated with other harmful habits, such as alcoholism and the use of injected drugs. Smoking was more frequent in cases with coexisting HIV infection, and those with a history of TB treatment. The prevalence of delayed diagnosis was significantly higher in smokers than in non-smokers. Pulmonary forms of TB, radiological cavitation, and positive smear-test results were more common in smokers, as was therapeutic non-compliance, and the need for directly observed treatment (DOT) administration and poorer treatment outcome. In contrast, we observed no differences between smokers and non-smokers in terms of single or multi-drug resistance, prescribed treatment, or clinical or radiological progression (Table 1).

**Table 1:** Characteristics of 5,846 tuberculosis patients. Factors related to smoking at the time of tuberculosis diagnosis at bivariate level

	Total	Non-smoker		Smoker		Bivariate analysis	
	N 5846	N 3546	61 %	N 2300	39 %	OR (95 % CI)	*P*-value
Sex							
Women	2082	1556	74,7	526	25,3	Ref	
Men	3626	1911	52,7	1715	47,3	2.65(2.36;2.99)	<0.001
Unknown	138	79	57,2	59	42,8	2.21(1.55;3.14)	<0,001
Age							
Unknown	138	118	85,5	20	15,5	Ref	
18–30 years	1627	1077	66,2	550	33,8	2.99(1.88;5.01)	<0,001
31–50 years	2405	1212	50,4	1193	49,6	5.77(3.65;9.61)	<0,001
> 50 years	1676	1139	68	537	32	2.76(1.74;4.62)	<0,001
Origin							
Immigrant	1941	1408	72,5	533	27,5	Ref	
Native	3905	2138	54,8	1767	45,2	2,18(1,94;2.46)	<0,001
Cohabitation status							
Group	651	431	66,2	220	33,8	Ref	
Alone	554	276	49,8	278	50,2	1,97(1,56;2,49)	<0,001
Homeless	100	25	25	75	75	5,84(3,65;9,63)	<0,001
Confinement	80	38	47,5	42	52,5	2,16(1,35;3,47)	0,001
With Family	4293	2683	62,5	1610	37,5	1,18(0,99;1,40)	0,067
Unknown	168	93	55,4	75	44,6	1,58(1,12;2,23)	0,01
Employment status							
Retired	978	792	81	186	19	Ref	
Active	3026	1794	59,3	1232	40,7	2,92(2.46;3,95)	<0.001
Unemployed	1481	730	49,3	751	50,7	4,38(3,63;5,30)	<0.001
Incapacity	110	59	53,6	51	46,4	3,68(2,44;5,53)	<0.001
Unknown	251	171	68,1	80	31,9	1,99(1,46;2,71)	<0.001
Alcohol							
No	4475	3079	68,8	1396	31,2	Ref	
Yes	1371	467	34,1	904	65,9	4,27(3.76;4,86)	<0,001
IDU							
No	5768	3532	61,2	2236	38,8	Ref	
Yes	78	14	17,9	64	82,1	7,15(4.12;13,4)	<0,001
HIV							
No	4547	2783	61,2	1764	38,8	1,10(0,96;1,27)	0,15
Yes	269	108	40,1	161	59,9	2,60(1,97;3,92)	0,001
Unknown	1030	655	63,5	375	36.4	Ref	
Diagnosis							
Specialits service	898	602	67	296	33	Ref	
Hospital Emergency Room	2916	1691	58	1225	42	1,47(1,26;1,73)	<0,001
Primere care	1106	660	59,7	446	40,3	1,37(1,14;1,65)	0,001
Other	754	494	65,5	260	34,5	1,07(0,87;1,31)	0,515
Unknown	172	99	57,6	73	42,4	1,5(1,07;2,09)	0,018
Prior Treatment							
No	5287	3245	61,4	2042	38,6	Ref	
Yes	438	224	51,1	214	48,9	1,52(1,25;1,85)	<0,001
Localization							
Extra-pulmonary	611	497	81,3	114	18,7	Ref	
Pulmonary	4149	2299	55,4	1850	44,6	3,50(2.84;4.35)	<0,001
Disseminated	301	198	65,8	103	34,2	2,27(1,66;3,10)	<0,001
Unknown	785	552	70,3	233	29,7	1,84(1,43;2,38)	<0,001
Radiology							
Normal	610	480	78,7	130	21,3	Ref	
Abnormal cavitary	1922	889	46,3	1033	53,7	4,28(3.47;5,32)	<0,001
Abnormal non cavitary	3114	2042	65,6	1072	33,4	1,94(1,58;2,39)	<0,001
Unknown	200	135	67,5	65	32,5	1,78(1,24;2,53)	0,002
Microbiology							
Culture (−)	1055	779	73,8	276	26,2	Ref	
Microscopy smear (+)	3144	1636	52	1508	48	2,60(2,23;3,04)	<0,001
Microscopy smear (+), culture (−)	1647	1131	68,7	516	31,3	1,29(1,08;1,53)	0,004
Single-drug resistance							
No	5458	3311	60,7	2147	39,3	Ref	
Yes	388	235	60,6	153	39,4	1,00(0,81;1,24)	0,968
Multi-drug resistance							
No	5797	3517	60,7	2280	39,3	Ref	
Yes	49	29	59,2	20	40,8	1,07(0,59;1,88)	0,827
Delayed diagnosis							
Unknown	343	226	65,9	117	34,1	Ref	
< 50 days	2777	1680	60,5	1097	39,5	1,26(1,00;1,60)	0,052
> 50 days	2726	1640	60,2	1086	39,8	1,28(1,01;1,62)	0,04
Treatment indicated							
Unknown	197	132	67	65	33	Ref	
3 drugs	2566	1521	59,3	1045	40,7	1,39(1,03;1,91)	0,032
4 drugs	2819	1732	61,4	1087	38,6	1,27(0,94;1,74)	0,119
Others	264	161	61	103	39	1,30(0,88;1,92)	0,186
Need for DOT							
Unknown	597	373	62,5	224	37,5	Ref	
No	4629	2834	61,2	1795	38,8	1,05(0,89;1,26)	0,555
Yes	620	339	54,7	281	45,3	1,38(1,10;1,74)	0,006
Clinical progression							
Unknown	569	354	62,2	215	37,8	Ref	
Improved	4860	2936	60,4	1924	39,6	1,08(0,90;1,19)	0,406
Stable	368	226	61,4	142	38,6	1,03(0,79;1,35)	0,805
Progressed	49	30	61,2	19	38,8	1,05(0,56;1,89)	0,884
Radiological progression							
Stable	981	626	63,8	355	36,2	Ref	
Improved	3750	2238	59,7	1512	40,3	1,19(1,03;1,38)	0,018
Progressed	36	17	47,2	19	52,8	1,97(1,00;3,89)	0,049
Unknown	1079	665	61,6	414	38,4	1,10(0,92;1,31)	0,307
Treatment outcome							
Unknown	520	324	62,3	196	37,7	Ref	
Correct	5079	3123	61,5	1956	38,5	1,04(0,86;1,25)	0,717
Incorrect	247	99	40,1	148	59,9	2,47(1,81;3,37)	<0,001

*OR* odds ratio

*CI* confidence interval

*IDU* injecten drug users

*HVI* humanimmunodeficiency virus

The multivariate analysis (Table 2) showed that the following factors were associated with smoking at the time of diagnosis in this cohort of TB patients: male gender (OR = 2.26,CI 1.97–2.60), being native Spanish (OR = 2.79,CI 2.40–3.24), alcohol consumption (OR = 2.85,CI 2.46–3.31), IDU (OR = 2.78,CI 1.48–5.52), poverty (OR = 1.99,CI 1.14–3.57), pulmonary forms of TB (OR = 1.61,CI 1.16–2.24), cavitary radiological patterns (OR = 1.99,CI 1.43–2.79) and positive smear-test results (OR = 1.39, CI 1.14–1.71).

**Table 2:** Characteristics of 5,846 tuberculosis patients. Factors related to smoking at the time of tuberculosis diagnosis at multivariate level

	Total	Non-smoker		Smoker		Multi-variate analysis.	
	N 5846	N 3546	61 %	N 2300	39 %	OR (95 % CI)	*P*-value
Sex							
Women	2082	1556	74,7	526	25,3	Ref	
Men	3626	1911	52,7	1715	47,3	2.26(1.97;2.60)	<0.001
Unknown	138	79	57,2	59	42,8	2.19(1.46;3.28)	<0.001
Age							
Unknown	138	118	85,5	20	15,5	Ref	
18–30 years	1627	1077	66,2	550	33,8	2.39(1.44;4,13)	<0.001
31–50 years	2405	1212	50,4	1193	49,6	3.45(2.09;5,94	<0.001
> 50 years	1676	1139	68	537	32	2.52(1.50;4.42	<0.001
Origin							
Immigrant	1941	1408	72,5	533	27,5	Ref	
Native	3905	2138	54,8	1767	45,2	2,79(2,40;3,24)	<0,001
Cohabitation status							
Group	651	431	66,2	220	33,8	Ref	
Alone	554	276	49,8	278	50,2	1,21(0,91;1,60)	0,174
Homeless	100	25	25	75	75	1,99(1,14;3,57)	<0.001
Confinement	80	38	47,5	42	52,5	1,70(0,98;2,97)	0,056
With Family	4293	2683	62,5	1610	37,5	0,89(0,72;1,11)	0,317
Unknown	168	93	55,4	75	44,6	1,35(0,89;2,04)	0,147
Alcohol							
No	4475	3079	68,8	1396	31,2	Ref	
Yes	1371	467	34,1	904	65,9	2,85(2,46;3,31)	<0,001
IDU							
No	5768	3532	61,2	2236	38,8		
Yes	78	14	17,9	64	82,1	2.78(1.48;5.52)	<0,001
HIV							
No	4547	2783	61,2	1764	38,8	0,81(0,34;2,07)	0,646
Yes	269	108	40,1	161	59,9	1,48(0,59;3,94)	0,412
Unknown	1030	655	63,5	375	36.4	Ref	
Prior Treatment							
No	5287	3245	61,4	2042	38,6	Ref	
Yes	438	224	51,1	214	48,9	1,12(0,89;2,04)	0,314
Localization							
Extra-pulmonary	611	497	81,3	114	18,7	Ref	
Pulmonary	4149	2299	55,4	1850	44,6	1,61(1,16;2,24)	<0,001
Disseminated	301	198	65,8	103	34,2	1,12(0,73;1,70)	0,59
Unknown	785	552	70,3	233	29,7	1,14(0,80;1,63)	0,458
Radiology							
Normal	610	480	78,7	130	21,3	Ref	
Abnormall cavitary	1922	889	46,3	1033	53,7	1,99(1,43;2,79)	<0,001
Abnormal non cavitary	3114	2042	65,6	1072	33,4	1,24(0,91;1,70)	0,167
Unknown	200	135	67,5	65	32,5	1,21(0,77;1,89)	0,4
Microbiology							
Culture (−)	1055	779	73,8	276	26,2	Ref	
Microscopy smear (+)	3144	1636	52	1508	48	1,39(1,14;1,71)	<0,001
Microscopy smear (+), culture (−)	1647	1131	68,7	516	31,3	1,01(0,82;1,24)	0,896

*OR* odds ratio

*CI* confidence interval

*IDU* injecten drug users

*HVI* humanimmunodeficiency virus

## Discussion

We observed a high prevalence of smoking among individuals diagnosed with TB in Spain between 2006 and 2013. Smoking was associated with male gender, being native Spanish, the consumption of other drugs, a precarious social position, more severe and more developed lung disease, and poorer treatment outcome than in non-smoking TB patients.

In this study, we found that 39.3 % of TB patients were regular smokers at the time of diagnosis, a much higher figure than that reported for the general population in Spain, 23.95 %, according to data from the National Health Survey 2011–2013 and it remained well above this value, and with little annual variation (Fig. 1); this level was similar to that published for Catalonia in 2002 (34.9 %) [12]. However, during the period of this study, we observed a steady decline in smoking prevalence in Spain, 26.44 % in 2006 to 23.95 % in 2013 [21], as a result of legislative changes and prevention programs [22]. Thus, the TB patients smokers may be resistant to general anti-smoking measures, possibly for social reasons or because of differences in its level of addiction to nicotine. This hinders smoking cessation in this group, and highlights the need for a different set of cessation strategies to those used in the general population.

In our sample, we found that men smoke significantly more than women; 62 % of men with TB were smokers, compared to 27.87 % of males in the general population during the same period. In comparison, the prevalence of smoking among female TB patients (25.3 %) was only 5 percentage points higher than in the general population (20.2 %). Worldwide, TB is known to be more common in men than in women. A study conducted in 22 countries with a high burden of TB found that smoking was a predictor of increased reporting of TB in males [23], suggesting that the differences in disease rates between sexes could be due to the higher prevalence of smoking in men, a pattern that persists in virtually all ethnic groups and countries [2].

While a third of the cases included in this study were foreigners, the prevalence of smoking was much lower in this group (27.5 %) than in native Spanish patients (45.2 %). Previous studies carried out in Spain show that the intensity of drug and alcohol consumption is lower in the immigrant population than in natives. This pattern has been attributed to cultural differences and economic difficulties that reduce consumption, and is maintained in immigrants with TB [24].

Smoking was also associated with situations of social precariousness, and its prevalence was significantly higher among individuals who live alone, in poverty, or in confinement, regardless of their age or employment status. Smoking was also associated with pathological alcohol consumption (65.9 %) and injected drug use (82.1 %). These risk factors are classically associated with TB in people at risk of social marginalization, as previously described in our setting [12, 24, 25]. The proportion of HIV co-infection was low in this sample (4.6 %), and HIV was associated with tobacco use in the bivariate but not multivariate analysis, possibly because of the low number of co-infected cases. The clinical presentation of TB in smokers was mainly pulmonary and disseminated, while isolated extra-pulmonary TB was rare in smokers. Radiologically, we also observed more extensive lesions in these patients, with more frequent cavitation and positive smear results at the time of diagnosis. These findings are similar to those reported by other authors, mentioned above, and reflect generally more serious and advanced disease [12, 13]. Several studies in animal models and humans have shown that exposure to tobacco smoke causes immunological changes, acting on alveolar macrophages by decreasing the production of TNF-α, IFN-γ, and mucociliary clearance, promoting disease progression [26–28], delaying sputum conversion and thereby extending the period of transmissibility [29]. In this sense, and in contrast to other studies [12], the observed delay in diagnosis was also higher in smokers, possibly because cough is a common symptom in these patients, and it may be difficult to perceive changes that alert the subject and motivate them to seek medical advice.

We did not find any significant differences between smokers and non-smokers in the presence of single or multi-drug resistance, or in the treatment initially indicated, although directly observed therapy was indicated more frequently in smokers than in non-smokers. Poorer treatment outcome was also more common among smokers. Therefore, smokers require closer monitoring and greater resources to ensure therapeutic compliance and the ultimate success of treatment, leading to increased healthcare spending. Other studies have also found a greater need for hospitalization, longer stays [12], and increased risk of relapse following treatment. A study in Taiwan found that people who smoke more than 10 cigarettes per day have twice the risk of relapse of non-smokers after proper treatment [14]. In our series, 7.5 % of patients had a history of previous illness, which was significantly with smoking.

Our study has some limitations. Our work can only show association between smoking and tuberculosis from an epidemiological point of view, and cannot show causality.

We did not quantify daily tobacco consumption, the number of years of smoking, type of tobacco used, or the intensity of passive smoking, which prevents us from evaluating a possible dose-response relationship. Only smoking status at the time of diagnosis was recorded, and we cannot determine if this persisted throughout the patient’s follow-up. Thus, it was not possible to assess the impact of smoking cessation on disease progression or the treatment outcome.

We could not collect systematic information on smoking cessation interventions by health professionals, since this survey depended solely on routine clinical practice and the experience of the individual medical teams in each of the participating centers. Some centers gave brief anti-smoking counseling, along with basic health education during each visit. This type of intervention has previously proven useful and feasible in other TB treatment programs [30], but is not included in local or national recommendations or regulations in our setting. The introduction of drug therapy for smoking cessation in patients with TB is still a pending task, given the limited clinical experience available [31]; few professionals feel prepared to advise their TB patients on smoking cessation [32], and cessation drugs are not widely accessible due to their high price because Spain are not subsidized by the National Health System and makes it impossible the access groups with economic difficulties as they are often patients with TB.

One of this study’s strong points is the large number of cases recruited representing 12 % of total cases reported in the regions that are part of the Spanish state during the study period [33] and the quality of the information, which allowed us to analyze trends in prevalence during the study period and to evaluate factors associated with smoking among TB patients.

## Conclusion

The prevalence of smoking among TB patients in Spain is high. Smokers with TB have a distinct sociodemographic, clinical, radiological and microbiological profile to non-smokers. A detailed understanding of the prevalence of smoking in our setting, as well as sociodemographic, clinical and developmental factors associated with smoking among TB patients is the first step towards designing effective strategies for control and monitoring, with the aim of improving the care of these patients, their clinical progression, and the treatment outcome.
